# Acute and Chronic Toxicity of Binary Mixtures of Bisphenol A and Heavy Metals

**DOI:** 10.3390/toxics10050255

**Published:** 2022-05-17

**Authors:** Jun Yang, Anqi Liao, Shulin Hu, Yiwen Zheng, Shuli Liang, Shuangyan Han, Ying Lin

**Affiliations:** 1 School of Biology and Biological Engineering, South China University of Technology, Guangzhou 510006, China; bijunyang@mail.scut.edu.cn (J.Y.); 202021049679@mail.scut.edu.cn (A.L.); 201921046830@mail.scut.edu.cn (S.H.); 201921047078@mail.scut.edu.cn (Y.Z.); shuli@scut.edu.cn (S.L.); syhan@scut.edu.cn (S.H.); 2 Guangdong Provincial Key Laboratory of Fermentation and Enzyme Engineering, South China University of Technology, Guangzhou 510006, China

**Keywords:** mixture toxicity, bisphenol A, heavy metal, joint effect, *Vibrio qinghaiensis* Q67

## Abstract

Bisphenol A (BPA) and heavy metals are widespread contaminants in the environment. However, the combined toxicities of these contaminants are still unknown. In this study, the bioluminescent bacteria *Vibrio qinghaiensis* Q67 was used to detect the single and combined toxicities of BPA and heavy metals, then the joint effects of these contaminants were evaluated. The results show that chronic toxicities of chromium (Cr), cadmium (Cd), lead (Pb), arsenic (As), mercury (Hg), nickel (Ni), and BPA were time–dependent; in fact, the acute toxicities of these contaminants were stronger than the chronic toxicities. Furthermore, the combined toxicities of BPA and heavy metals displayed BPA + Hg > BPA + Cr > BPA + As > BPA + Ni > BPA + Pb > BPA + Cd in the acute test and BPA + Hg > BPA + Cd > BPA + As > BPA + Cd in the chronic test, which suggested that the combined toxicity of BPA and Hg was stronger than that of other mixtures in acute as well as chronic tests. Additionally, both CA and IA models underestimated the toxicities of mixtures at low concentrations but overestimated them at high concentrations, which indicates that CA and IA models were not suitable to predict the toxicities of mixtures of BPA and heavy metals. Moreover, the joint effects of BPA and heavy metals mainly showed antagonism and additive in the context of acute exposure but synergism and additive in the context of chronic exposure. Indeed, the difference in the joint effects on acute and chronic exposure can be explained by the possibility that mixtures inhibited cell growth and luminescence in chronic cultivation. The chronic toxicity of the mixture should be considered if the mixture results in the inhibition of the growth of cells.

## 1. Introduction

BPA is an important industrial chemical that has been widely used as the monomer of polycarbonate plastics and epoxy resins. Materials containing BPA appear in our daily life, especially in food contact materials, such as plastic bottles, cups, plates, goblets, and storage containers [1,2]. Heavy metals, which are natural substances in the Earth’s crust, are widely spread in environment and foods [3]. The increasing demand for BPA has resulted in its accumulation in the environment [4,5,6]. BPA has been detected in packaged foods, drinking water, dust, sewage sludge, urine, serum, etc. [2,7]. A mean value of 8.99 ng/g BPA was tested in 45 vegetable samples [8]. Previous studies have shown that the concentration of BPA reached 174.6 ug/mL in a river sample [9] and 0.98 mg/L BPA was measured in drinking water [10]. Significantly, the concentration of BPA ranged from 0.1 to 3.9 ng/mL in 3021 human milk samples in 50 studies [11]. Therefore, the pollution of BPA has become serious. Furthermore, BPA and heavy metals widely co–exist in the environment and in foods. The toxicities of their mixtures must therefore be investigated.

The single toxicities of BPA and heavy metals have been well studied. BPA is not only well–known as an endocrine disrupter, but it also exhibits genotoxic activity [12] and reproductive [13,14], nervous [15,16], developmental [17,18], and immune toxicity [19,20]. Heavy metals can accumulate in the human body because of their long half–life, which results in diseases in target organs, such as the brain, kidney, and liver. [3]. Considering the long experimental time and poor repeatability for animal tests, especially the 3R principles regarding the ethical use of animals [21,22,23], a rapid and low–cost bioassay based on bioluminescent bacteria has been developed. Bioluminescent bacteria can rapidly respond to contaminants within a short time, and the light emission decreases with the increase in the concentration of contaminants [24,25]. To date, the acute toxicity of BPA evaluated by bioluminescent bacteria is lacking; however, the toxicities of heavy metals to bioluminescent bacteria are well studied in the environmental field. Most natural bioluminescent bacteria are isolated from the ocean, including four genera, namely *Photobacterium*, *Photorhabdus*, *Shewanella*, and *Vibrio* [25,26]. *Photobacterium phosphoreum* T3, *Vibrio fischeri,* and *V*. *qinghaiensis* Q67 are widely used to detect the acute toxicity of contaminants [25,27]. The acute toxicities of heavy metals, such as chromium, cadmium, copper, mercury, lead, and zinc were tested using *V. fischeri*, *V. qinghaiensis* Q67, and *P. phosphoreum* [28,29,30]. Indeed, it is known that high concentrations of NaCl (2–3%, *w*/*v*) impact the toxicities of contaminants, especially heavy metals [31,32,33]. Therefore, Q67 is suitable to measure the toxicities of heavy metals because it is the only strain isolated from freshwater so far. Thus, a lower concentration of NaCl (0.85%, *w*/*v*) needs to be added to samples [28].

In recent decades, binary- and ternary-mixture toxicities regarding BPA and other chemicals have been studied. However, the toxicities of mixtures of BPA and other chemicals have mainly focused on BPA and other endocrine disrupters (Eds) [16,34,35]. Studies regarding the combined toxicities of BPA and its analogues are increasing because BPA was replaced by its analogues due to its adverse effect [2,36,37,38]. In addition, the toxicities of mixtures of BPA and TiO_2_ [15,17,39], Pb [16], and Cd [40] have been investigated using different types of wildlife; however, no clear synergism, additive, or antagonism effects were obtained in these works. The mixture toxicities of BPA and phthalates were studied using different organisms [41,42,43,44]. The joint effect of BPA and dibutyl phthalate was investigated, and the results showed that the co–exposure of these contaminants resulted in increased cytotoxicity, oxidative stress, and genotoxicity [40]. The mixtures of BPA and diethylhexyl phthalate (DEHP) and dibutyl phthalate (DBP) when exposed to human amniotic fluid reduced INSL3/RXFP2 signaling, which revealed an antagonist effect [45]. The antagonism effect of BPA and DEHP was observed in juvenile rats [46]. In addition, the joint effects of BPA and its analogues BPS, BPAF, BPB, and BPF were assessed by a highly sensitive micro–biosensor, and additive and synergistic effects were observed [47]. It is worth noting that *V. fischeri* was used to assess the joint effects of bisphenols and plasticizers and pharmaceuticals, and synergistic and antagonistic effects were observed [37]. Nonetheless, studies regarding the toxicities of mixtures of BPA and heavy metals are lacking, and their joint effects are still unknown. Bioluminescent bacteria have rarely been used to measure toxicities of mixtures of BPA and other contaminants until now. The rapid and low–cost approach based on bioluminescent bacteria should be used to estimate the joint effects of BPA and heavy metals in the environmental field.

In this study, the toxicities of binary mixtures of BPA and heavy metals were evaluated using bioluminescent bacteria *V. qinghaiensis* Q67. Further, the conventional models, CA and IA, were used to predict the toxicities of the mixtures. Moreover, the joint effects of BPA and heavy metals were assessed using the toxicity–unite method. This work proposes the application of Q67 for the detection of the toxicities of mixtures of BPA and other contaminants. Q67, which was isolated from Qinghai Lake in China, was the only strain isolated from the freshwater, and it might be the appropriate model from the aquatic system. The toxicity evaluated by Q67 might indicate the real toxicity of samples, which would contribute to ecotoxicology and risk assessment.

## 2. Materials and Methods

### 2.1. Bacterial Strains and Contaminants

The bioluminescent bacteria used in this work was *V*. *qinghaiensis* Q67. The freeze–dried Q67, used for the detection of the acute toxicity alone and in the mixture, was prepared according to a previous study [48]. The contaminants used in this study were bisphenol A (BPA), K_2_Cr_2_O_7_, Cd(NO_3_)_2_, ZnSO_4_·7H_2_O, Pb(NO_3_)_2_, arsenic (As), mercury (Hg), and nickel (Ni). BPA (GC, purity > 99%) was purchased from Macklin (Shanghai, China). All the other chemicals were purchased from Aladdin (Shanghai, China). All the concentrations of contaminants used in this work are listed in Table S1. It is worth mentioning that an aqueous BPA solution was prepared and compared with BPA dissolved in methanol. BPA was dissolved in deionized water at 75 °C for 2 h; then, the quantities of BPA dissolved in water and methanol at the same concentrations were compared using the e2695–series high–performance liquid chromatography (HPLC) system (Waters, Milford, MA, USA) [49]. Briefly, the column was ZORBAX Eclipse XDB–C18 (Agilent Technology, Santa Clara, CA, USA), the liquid phase was 60% (*v*/*v*) acetonitrile, the injection volume of the sample was 20 μL, the temperature was kept at 30 °C, and the detection wavelength was 276 nm.

### 2.2. Culture Medium and Culture Conditions

The fresh cultured *V*. *qinghaiensis* Q67 was used for the measurement of chronic toxicity. The medium for the Q67 contained tryptone (5 g/L), yeast extract (5 g/L), MgCl_2_ (3.2 g/L), KBr (0.2 g/L), CaSO4 (0.1 g/L), KCl (4 g/L), NaCl (4 g/L), and glycerol (3 mL/L), with the pH adjusted to 8.5 [50]. *V*. *qinghaiensis* Q67 was cultured at 20 °C for 12 h with 180 rpm shaking; then, the harvested fresh culture was used to test the chronic toxicity.

### 2.3. Single Toxicity

The detection of acute and chronic single toxicity was performed according to the method used in a previous study [51,52]. For the test of the acute toxicity, the freeze–dried Q67 was resuscitated in a 0.85% NaCl solution for 15 min; then, 10 μL of resuscitated Q67 was transferred into a 150 μL contaminant solution. The contaminant solution was diluted using 0.85% NaCl over a range of concentrations. In terms of the chronic toxicity, 90 μL contaminant solution and 90 μL of 2–fold culture medium were first mixed; then, 20 μL of the fresh culture of Q67 was transferred into the last mixture. Additionally, the mixture was oscillated uniformly and incubated at 23 °C for 6 or 12 h. Finally, the bioluminescence of the mixtures was recorded by a SYNERGY H1 microplate reader (BioTek, Winooski, VT, USA). The arrangement of control and samples in a 96–well plate are shown in Figure S1. The inhibition ratio of the luminescence was calculated according to Equation (1). The concentration–inhibition data were fitted by the logistic (Equation (2)) and the dose–response model (Equation (3)) [52,53].

(1) $$I=\frac{L_{C}-L_{S}}{L_{C}}\times 100\%$$

where *I* is the inhibition ratio of luminescence. *L_C_* and *L_S_* indicate the relative luminescence unit of control and contaminants solution, respectively.

(2) $$I=A_{2}+\frac{A_{1}-A_{2}}{1+{\left(C/C_{0}\right)}^{P}}$$

(3) $$I=A_{2}+\frac{A_{2}-A_{1}}{1+10^{\left(logC_{0}-C\right)P}}$$

where *A*_1_ and *A*_2_ are the bottom and top values, respectively, of the inhibition ratio. *C* is the concentration of tested samples, and *C*_0_ indicates the value of *C* at 50% of the inhibition ratio of luminescence. *P* is the parameter of slope for the concentration–inhibition curve.

### 2.4. Mixture Toxicity

The toxicities of mixtures of BPA and heavy metals were analyzed. The test of the toxicity of the binary mixture was carried out using the same method as that for single toxicity. Based on the *EC*_50_ values of individual contaminants, the mixture toxicities of BPA and Cr, Pb, Hg, and Ni at equitoxic ratios were detected, while BPA and Cd and As at non–equitoxic ratios were prepared at 1:10^−1.5^, 1:10^−1^, 1:10^−0.5^, 1:1, 10^0.5^:1, 10^1^:1, and 10^1.5^:1; then, the mixture toxicities were tested. The concentrations of individual contaminants in stocked mixtures are listed in Table S2. Stocked mixtures were diluted for further tests. The toxic unit of the mixture (*TU_mix_*) was used to evaluate the joint effects of mixtures, which were calculated using Equation (4). According to a previous study, 0.8 < *TU* < 1.2 was considered simple additivity, while *TU* < 0.8 revealed synergism; additionally, *TU* > 1.2 indicated antagonism [54,55].

(4) $$TU_{mix}=\frac{C_{A}}{EC_{50A}}+\frac{C_{B}}{EC_{50B}}$$

where *C_A_* and *C_B_* are the individual concentrations of *A* and *B*, respectively, in a mixture that inhibits 50% of luminescence. *EC*_50*A*_ and *EC*_50*B*_ are effective concentrations of individual contaminants at 50% inhibition according to single toxicity. 

### 2.5. Prediction of Mixture Toxicity

The quantitative structure–activity relationship (QSAR), independent action (IA), concentration addition (CA), and integrative models of the CA and IA such as two–stage prediction (TSP) are widely used models to predict the toxicity of mixtures. According to the different toxic modes of action, two conventional models are used to predict the joint effect of mixtures. One is independent action (IA, different mode of action, MOA), and the other is concentration addition (CA, similar to MOA) [56]. In this study, BPA and heavy metals indicate different toxic action modes, and both CA and IA were used to predict the mixture of toxicities. The mathematical functions of CA and IA are expressed as Equations (5) and (6), respectively:

(5) $$EC_{x,m}={\left(\frac{P_{A}}{EC_{x,A}}+\frac{P_{B}}{EC_{x,B}}\right)}^{-1}$$

where *EC*_*x*,*m*_, *EC*_*x*,*A*_, and *EC*_*x*,*B*_ are the concentrations of the mixtures; *A* and *B* are the decreases in the *x*% luminescence, respectively; and *P_A_* and *P_B_* correspond the concentration ratios of *A* and *B* in the mixture.

(6) $$E\left(C_{M}\right)=1-\left(1-E\left(C_{A}\right)\right)\times \left(1-E\left(C_{B}\right)\right)$$

where *E*(*C_M_*) is the toxic effect of the mixture, and *E*(*C_A_*) and *E*(*C_B_*) are the toxic effects of *A* and *B* in the individual toxicity tests, respectively.

### 2.6. Statistical Analysis

The statistical analysis was performed using Origin 8.5 and IBM SPSS 26.0 software. The difference between the acute and the chronic toxicity was analyzed, and *p* < 0.05 was considered statistically significant. The concentration–inhibition data were fitted using Origin 8.5, and the qualities of developed models were evaluated by the number of points, degrees of freedom, reduced chi–sqr, residual sum of squares, and R–squared (R^2^).

## 3. Results

### 3.1. Single Toxicity of BPA and Heavy Metals

**Figure 1:**
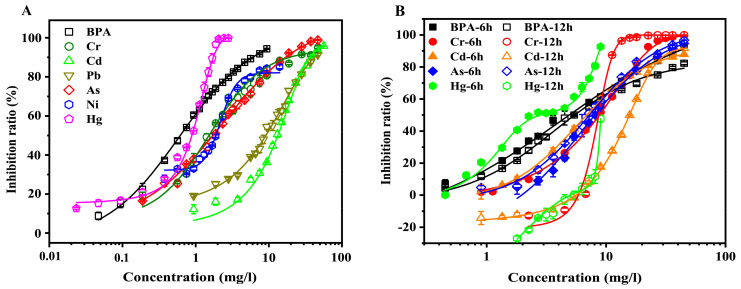
The acute (**A**) and chronic (**B**) single toxicity of BPA and Cr, Cd, Pb, As, Hg, and Ni. The concentration–inhibition data were fitted using the logistic and dose–response models. The error bars indicate the standard deviations from three independent experiments.

Before the examination of the binary–mixture toxicities of BPA and heavy metals, the single toxicities of BPA, Cr, Cd, Pb, As, Hg, and Ni to Q67 were tested (Figure 1). Considering that the acute toxicity of the aqueous BPA solution toward bioluminescent bacteria was detected for the first time, the quantities of solutions of aqueous BPA and BPA dissolved in methanol at the same concentrations were tested using HPLC and then compared. The results showed that the aqueous BPA and methanol dissolved BPA solutions exhibited similar retention times and peak areas (Figure S2). In terms of toxicity detection, most of these concentration–inhibition data could be fitted by logistic and dose–response models. However, the concentration–inhibition data of Hg were fitted better than others by the bi–dose–response model. These models suggested good quality, and the values of R^2^ were over 0.99 for all fitting models. The reduced chi–sqr values ranged from 1.57 to 30.85. The EC_50_ value of each contaminant was calculated by the fitting function, which indicated the toxicity of each contaminant.

For the acute toxicity (Figure 1A), BPA and Cd displayed the strongest and weakest toxicity to Q67, respectively. The EC_50_ values of these contaminants ranged from 0.62 to 13.27 mg/L. The toxicities of these contaminants were in the following order: BPA > Hg > Cr > As > Ni > Pb > Cd. In the case of chronic toxicity, Pb and Ni revealed no obvious toxicity effects to Q67 in the range of the experimental concentration (data not shown). In addition, the chronic toxicity of these contaminants suggested a time–dependent nature (Figure 1B). The chronic toxicities of BPA, Cr, Cd, and Hg decreased with the increase in exposure time. All the concentration–inhibition data fit well with the logistic model for 6 h exposure but not for 12 h exposure; thus, the exposure time for chronic exposure was 6 h in other studies. The EC_50_ values of these contaminants (6 h exposure time) ranged from 2.81 to 8.79 mg/L. The chronic toxicities of these contaminants were as follows: Hg > BPA > Cd > As > Cr. The difference in the acute and chronic toxicities of these contaminants was significant (*p* < 0.05). 

### 3.2. Toxicities of Binary–Mixtures of BPA and Heavy Metals

**Figure 2:**
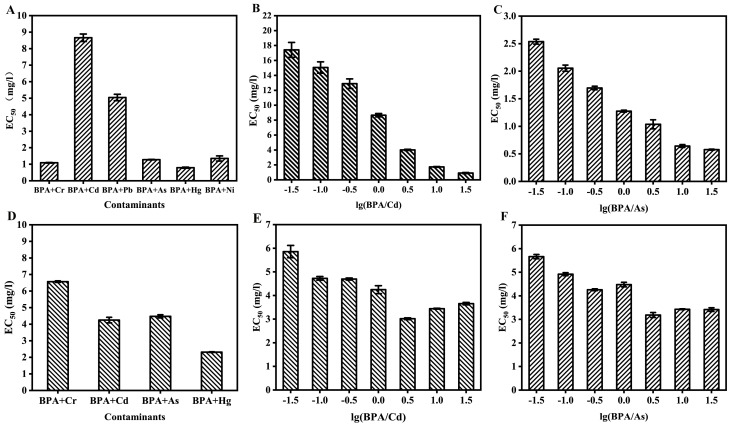
Toxicities of mixtures of BPA and heavy metals. (**A**) BPA and heavy metals at equitoxic ratios in acute toxicity tests; (**B**,**C**) BPA and Cd, BPA, and As at non–equitoxic ratios in the acute toxicity test, respectively; (**D**) BPA and heavy metals at equitoxic ratios in the chronic toxicity test; (**E**,**F**) BPA and Cd, BPA, and As at non–equitoxic ratios in the chronic toxicity test, respectively. The error bars indicate the standard deviations from three independent experiments.

Based on the single toxicities of these contaminants, the toxicities of binary mixtures of BPA and heavy metals were analyzed at equitoxic and non–equitoxic ratios. Concentration–inhibition data were fitted using same mathematic models as the single toxicity test. For all models developed in this work, the values of R^2^ (all over 0.99) and reduced chi–sqr (ranged from 0.70 to 15.86) indicated the good fits. In terms of acute toxicity of the mixtures, the EC_50_ of these binary mixtures revealed that their toxicities were as follows: BPA + Hg > BPA + Cr > BPA + As > BPA + Ni > BPA + Pb > BPA + Cd at equitoxic ratios (Figure 2A), which was the same as the single toxicities of heavy metals. In order to validate the toxicities of mixtures of BPA + Cd and BPA + As, the toxicities of mixtures of BPA + Cd and BPA + As at non–equitoxic were measured (Figures S3 and S4). The EC_50_ values of BPA + Cd ranged from 0.91 to 17.42 mg/L. Indeed, the toxicities of mixtures of BPA + Cd decreased with the increase in the ratio of lg(BPA/Cd) (Figure 2B), and the same conclusion was obtained from BPA + As (Figure 2C). These results indicate that the toxicities of mixtures of BPA + Cd and BPA + As were affected by the ratio of Cd and As in the mixture, respectively. 

In the case of chronic mixture toxicity, the toxicities of these mixtures were shown to be BPA + Hg > BPA + Cd > BPA + As > BPA + Cr at equitoxic ratios (Figure 2D and Figure S5), which is the same as the single toxicities of Hg, Cd, As, and Cr. Furthermore, toxicities of mixtures of BPA + Cd and BPA + As at non–equitoxic ratios were measured (Figures S6 and S7). The EC_50_ values of BPA + Cd at non–equitoxic ratios ranged from 3.02 to 5.86 mg/L. When the ratio of lg(BPA/Cd) was under 0, toxicities of mixtures of BPA + Cd decreased with the ratio of lg(BPA/Cd) increasing, while it increased with the increase in the ratio of lg(BPA/Cd) when the ratio of lg(BPA/Cd) was over 0 (Figure 2E); the same conclusion could be obtained from BPA + As (Figure 2F). The differences between the acute and chronic toxicities of mixtures of BPA + Cr, BPA + Cd, BPA + As, and BPA + Hg were statistically significant at equitoxic ratios as well as non–equitoxic ratios (*p* < 0.05).

### 3.3. Toxicities of Mixtures of BPA and Heavy Metals Predicted by CA and IA Models

**Figure 3:**
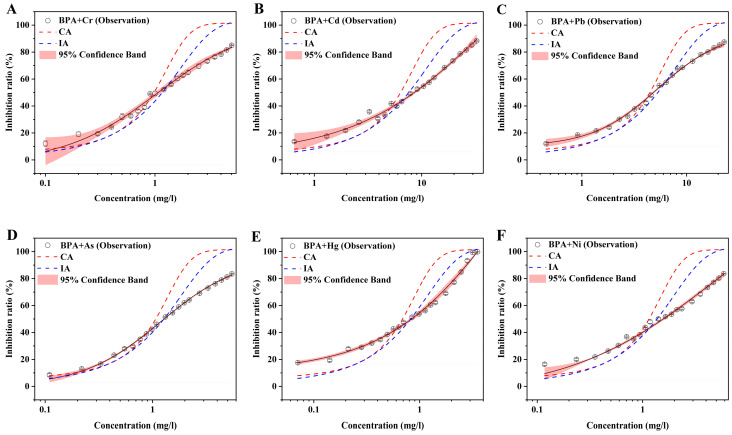
Acute toxicity of mixtures of BPA and Cr (**A**), Cd (**B**), Pb (**C**), As (**D**), Hg (**E**), and Ni (**F**) compared with the predicted value by the CA and IA models. The black circles are the experimental mean values of the toxicity of mixtures of BPA and heavy metals, and the black solid line is the fitting curve using the mathematical mode. The red shadow indicates the 95% confidence band. The red and blue dotted lines suggest the values predicted by the CA and IA models, respectively. The error bars indicate the standard deviations from three independent experiments.

According to previous studies, the IA model was usually used to predict the mixture toxicity of a mixture that exhibited dissimilar MOA. BPA and heavy metals seemed to display dissimilar MOAs, which should be validated. Therefore, both CA and IA models were used to predict the toxicities of these mixtures. In this study, the acute toxicities of mixtures of BPA and heavy metals were measured at equitoxic ratios, and then the observed experimental toxicities were compared with the values predicted by the CA and IA models (Figure 3). The concentration–inhibition data were fitted using logistic and dose–response models, and the quality of all developed models was good (R^2^ > 0.99). Due to the slow increase in the single toxicity of BPA with the increase in concentration, the mixture toxicities of BPA and heavy metals showed a similar relationship. However, the mixture of BPA + Hg revealed stronger toxicity than the other mixtures (Figure 3E). The mixture toxicity of BPA and Hg increased rapidly with the increase in concentration. In addition, the results showed that both CA and IA models underestimated the toxicity for all binary mixtures at low concentrations but overestimated the toxicity at high concentrations of the mixtures. Indeed, the value predicted by the IA model was lower than that predicted by the CA model, which was better close to the observed value. However, the IA model overestimated the toxicity of the mixture, and hence neither the CA nor the IA model was suitable to predict the toxicity of mixtures of BPA and heavy metals. 

### 3.4. Joint Effects of BPA and Heavy Metals

**Figure 4:**
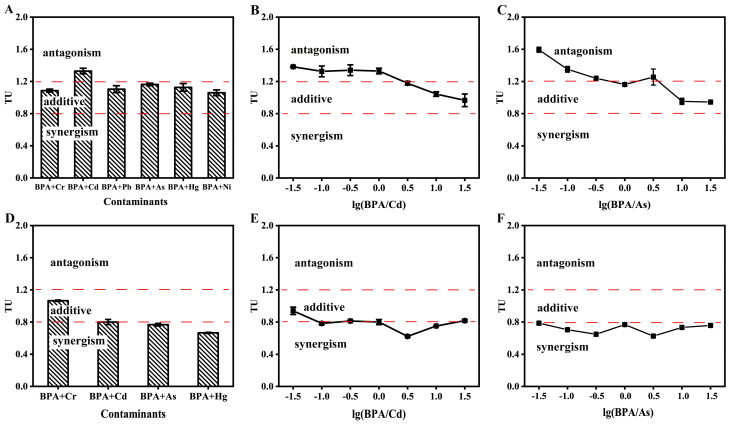
Joint effect of BPA and heavy metals. (**A**–**C**) BPA and heavy metals at equitoxic or non–equitoxic ratios in the acute toxicity test; (**D**–**F**) BPA and heavy metals at equitoxic or non–equitoxic ratios in a chronic toxicity test. The error bars indicate the standard deviations from three independent experiments.

According to the toxicities of mixtures of BPA and heavy metals, the EC_50_ values of mixtures (EC_50m_) were calculated using Equations (2) and (3). Based on the EC_50_ values of the mixtures, the TUs of mixtures were obtained using Equation (4). In the case of the acute toxicity of the mixtures, the joint effects of BPA and heavy metals exhibited simple additive and antagonisms, and the TU values of mixtures ranged from 1.06 to 1.33 (Figure 4A). BPA and Cd showed an antagonistic effect at the equitoxic ratio (the TU of the mixture was 1.33), whereas simple additive effects were observed among BPA and other heavy metals at the equitoxic ratio. In addition, according to the EC_50_ values of BPA + Cd and BPA + As at a non–equitoxic ratio, the joint effects of BPA + Cd and BPA + As exhibited antagonisms and simple additives (Figure 4B,C). The TU values of BPA + Cd at a non–equitoxic ratio ranged from 0.97 to 1.38, and the values decreased with the increase in the ratio of BPA in mixtures. The same conclusion could be drawn for BPA + As, and the range of EC_50_ values was from 0.94 to 1.59.

In the case of the chronic toxicities of mixtures, the joint effects of BPA and heavy metals indicated a synergistic and additive effect (Figure 4D). The TU values of BPA + Cr, BPA + Cd, BPA + As, and BPA + Hg were 1.07, 0.80, 0.77, and 0.67, respectively, at an equitoxic ratio. The joint effect of BPA and Cr suggested an additive effect, while the others revealed a synergism. Meanwhile, the joint effects of BPA + Cd and BPA + As suggested synergistic and additive effects at non–equitoxic ratios. The TU values of BPA + Cd ranged from 0.62 to 0.94, while the TU values of BPA + As were shown to range from 0.63 to 0.79. These results suggest that the joint effects of BPA and heavy metals between acute and chronic exposure were different. The joint effects of BPA and heavy metals on acute exposure were shown to be antagonisms and additives, whereas additive effects and synergism were obtained with chronic exposure.

### 3.5. Mechanism of the Synergistic Effects of BPA and Heavy Metals on V. qinghaiensis Q67

**Figure 5:**
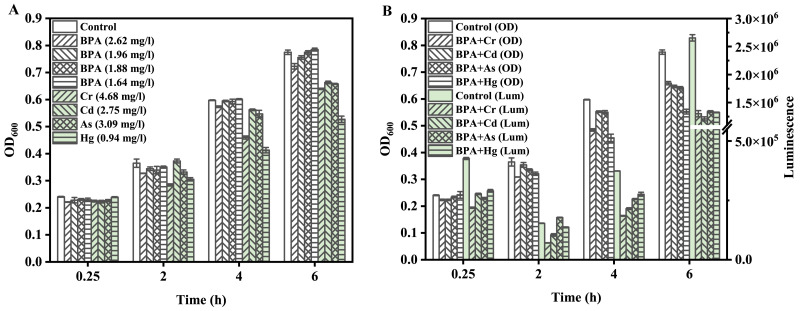
The impact of the mixtures of BPA and heavy metals (equitoxic ratio) on the growth and luminescence of *V. qinghaiensis* Q67. The concentrations of mixtures used in this work are the EC_50_ values of the mixtures in the test of the chronic toxicity of the mixtures, and the concentrations of BPA and heavy metals correspond to their individual concentrations in mixtures. The error bars indicate the standard deviations from three independent experiments. Impact of the individual contaminant on growth (**A**). The impact of mixtures on the growth and luminescence (**B**).

In terms of the joint effects, BPA and heavy metals exhibited different effects between the acute and the chronic test. For example, the joint effects of BPA and Cd showed antagonistic and additive effects for the acute test at non–equitoxic ratios but synergistic and additive effects for the chronic test. To investigate whether the synergistic effect was due to the inhibition of growth and luminescence, the impact of the contaminants on cell growth and luminescence was tested. The concentrations of mixtures corresponded to the EC_50_ values from the test of the mixtures’ toxicities, and the concentrations of BPA and heavy metals corresponded to their individual concentrations in mixtures. The final concentrations of BPA + Cr, BPA + Cd, BPA + As, and BPA + Hg in mixtures were 6.57, 4.25, 4.48, and 2.31 mg/L, respectively. The results showed that BPA barely inhibited cell growth, but heavy metals significantly inhibited that. All the binary mixtures inhibited the growth of Q67 (Figure 5). Meanwhile, the luminescence inhibition ratio of mixtures was close to 50% after 6 h exposure but below 50% at earlier cultivation times (0.25, 2, and 4 h) except for BPA + Cr. The luminescence inhibitions of BPA and Cr were constantly close to 50% throughout the exposure time. In other words, the toxicities of mixtures of BPA and Cd, As, and Hg were shown to be time–dependent but BPA + Cr was not. It is worth mentioning that the joint effect of BPA and Cr displayed simple additive effects not only for 15 min exposure in the acute test but also for 15 min, and 2, 4, and 6 h exposure in the chronic test.

## 4. Discussion

BPA and heavy metals are widely spread in the environment. BPA is well known as an endocrine disrupter that appears in daily human life, even in human milk [11]. This study indicates that BPA exhibits a stronger acute toxicity than heavy metals. Studies regarding the toxicity of mixtures of BPA and other contaminants have mainly focused on BPA and its analogues, EDs. However, works on the toxicities of mixtures of BPA and heavy metals are lacking. The co–exposure of BPA and heavy metals in the environment should be further studied. In this study, the toxicities of binary mixtures of BPA and heavy metals were investigated using bioluminescent bacteria *V. qinghaiensis* Q67. The results indicated that BPA and heavy metals displayed an antagonistic and an additive effect in the context of acute exposure but synergistic and additive joint effects in the contexts of chronic exposure. Compared with other bioluminescent bacteria, such as *V. fischeri* and *P. phosphoreum* T3, Q67 is the only strain isolated from freshwater so far. Thus, the low concentration of NaCl added to samples decreased the impact of NaCl on the toxicities of heavy metals [31,32,33]. Q67 is therefore a suitable bioluminescent bacterial strain to detect the mixture toxicities of BPA and heavy metals. The results from bioluminescent bacteria might not be repeated in animal tests, but they could be considered a rapid screening approach used in the environmental field, especially in aqueous samples.

Studies regarding the mixture toxicities of BPA and heavy metals have been conducted in the past. The prenatal co–exposure of BPA and Pb has been associated with neurotoxicity, but the interaction of BPA and Pb should be further studied [16]. In addition, the toxicity of mixtures of BPA and Cd was investigated in HepG 2 cells, and the results showed that the co–exposure of BPA and Cd enhanced the cytotoxicity, oxidative stress, and genotoxicity, indicating an additive and synergistic effect [40]. The additive and synergistic effects of BPA and Cd could be also obtained from a chronic test in this study. However, the acute joint effects of BPA and Cd were shown to be the antagonism and additive in this work. The chronic joint effect of BPA and Cd toward Q67 in this study was similar to a previous study. In fact, the detection of the toxicity of mixture contaminants to higher organisms and cells was often exhibited in the long term, which was similar to the chronic toxicity test of contaminants toward Q67. Otherwise, there are some similar studies that should be compared with this work. In one study, toxicities of mixtures of phthalate esters and Cd to *V. qinghaiensis* Q67 were investigated, and the results showed that mixtures of phthalate esters and Cd showed additive effects [57]. The EC_50_ value of Cd in that study (18.8 mg/L) was higher than in our study (13.274 mg/L). Further, the synergistic and antagonistic effects of the ternary mixtures of BPA, diethyl phthalate (DEP), and diglycidyl ether (BADGE) to *V. fischeri* were confirmed; however, BPA and other chemicals were dissolved in methanol in that study [37]. Indeed, methanol can affect the luminescence of bioluminescent bacteria. Therefore, the aqueous BPA solution was applicable for acute toxicity detection using bioluminescent bacteria. 

The quantitative structure–activity relationship (QSAR) model, CA and IA models, and integrated CA and IA models such as TSP (two–stage prediction) have been used to predict the toxicity of mixtures of contaminants [56]. The CA model is usually used to predict the mixtures with the same MOA but IA with different MOA. The toxicities of mixtures of Cd, Pb, and Mn were predicted well by CA models [58]. However, the observed toxicities of these mixtures were often lower or higher than the predicted value. This was illustrated by the toxicities of mixtures of zinc oxide nanoparticles and chemicals with different MOAs, and neither CA nor IA models were suitable to predict the toxicities of mixtures of these contaminants [52]. Therefore, the prediction of the toxicity of mixtures in silicon was significant but should combined with the actual observed toxicity in experiments.

The difference in the joint effect of acute and chronic exposure was investigated (Figure 5), and the difference can be explained by mixtures inhibiting the growth of bioluminescent bacteria in 6 h long–term exposure. The luminescence in samples containing mixtures of BPA and heavy metals decreased by 50% relative to the control after 6 h of incubation; however, the biomass of these samples was lower than the control. In other words, the stronger inhibition of luminescence (over 50% ratio) should appear when the biomass of the samples equals the control. Consequently, mixtures of BPA and heavy metals inhibiting both cell growth and luminescence resulted in an antagonistic effect in acute exposure, which converted to a synergistic effect in the context of chronic exposure. The ecotoxicity of BPA to different classes of creatures, such as annelids, mollusks, crustaceans, insects, fish, and amphibians was investigated in past decades [20]. Additionally, such toxicity was also illustrated by BPA inhibiting algal and cyanobacterial growth [59,60]. Thus, it is reasonable that BPA inhibits the growth of bioluminescent bacteria. The effect of heavy metals on different creatures has been studied. Hg, Cd, Cu, Zn, Pb, and Cr affect the survival of ciliates [61]. Meanwhile, the influence of metals on the populations of microorganisms has been illustrated in the context of long–term exposure [62]. In this study, similar results were obtained in the chronic toxicity test (6 h exposure) (Figure 5A). Indeed, the mixture also inhibited the growth of Q67 (Figure 5B). Therefore, the joint effect of BPA and heavy metals revealed a synergism in the context of chronic detection. However, the joint effect of BPA and Cr exhibited an additive effect in both an acute and chronic test. In addition, the toxicities of mixtures of BPA and Cd, As, and Hg were shown to be time–dependent, but BPA and Cr were not, in the context of chronic exposure. BPA and Cr might present the same MOA, but this needs to be validated by further studies.

## 5. Conclusions

The acute and chronic toxicities of BPA and heavy metals, individually and in mixtures, were determined using bioluminescent bacteria *V. qinghaiensis* Q67 isolated from freshwater. The results showed that single chronic toxicities of BPA and heavy metals were time–dependent, and the toxicities of these contaminants were shown to be stronger in acute exposure than in chronic exposure, except for Cd. Furthermore, neither CA nor IA models were suitable for the prediction of the mixture toxicities of BPA and heavy metals. Moreover, the acute joint effects of BPA and heavy metals mainly displayed antagonistic and additive effects, whereas the chronic joint effects of these mixtures mainly exhibited synergistic and additive effects. Indeed, the mixtures inhibiting both cell growth and luminescence resulted in antagonistic effects in the context of acute exposure, shifting to a synergistic effect in the context of chronic exposure.

## Data Availability

The dataset is available upon the request of the corresponding authors.

## Supplementary Materials

The following supporting information can be downloaded at: https://www.mdpi.com/article/10.3390/toxics10050255/s1, Figure S1: Arrangement of control and test samples in 96–well microplate. Gray solid circles are the control, and the blue solid circles are test samples. Each concentration of sample locates in four wells in same column; Figure S2: Comparison of aqueous BPA solution and methanol dissolved BPA using HPLC. Black line, methanol dissolved BPA solution. Red line, aqueous BPA solution; Figure S3: Observed acute mixture toxicity of BPA and Cd at non–equitoxic ratio; Figure S4: Observed acute mixture toxicity of BPA and As at non–equitoxic ratio; Figure S5: Observed chronic mixture toxicity of BPA and heavy metals at equitoxic ratio; Figure S6: Observed chronic mixture toxicity of BPA and Cd at non–equitoxic ratio; Figure S7: Observed chronic mixture toxicity of BPA and As at non–equitoxic ratio; Table S1: Concentration of individual contaminant used in single toxicity test; Table S2: Concentrations of individual contaminants in stocked mixtures.
