# Supplementary material for: Acute and Chronic Toxicity of Binary Mixtures of Bisphenol A and Heavy Metals
Source: Toxics. 2022 May 17;10(5):255. doi: 10.3390/toxics10050255 (PMC9145676; doi:10.3390/toxics10050255)
Supplement: Supplementary file 1 [file toxics-10-00255-s001.zip › toxics-1702140-supplementary.pdf]

toxics

MDPI

Supplementary Materials: Acute and Chronic Toxicity of Binary Mixtures of Bisphenol A and Heavy Metals

Jun Yang, Anqi Liao, Shulin Hu, Yiwen Zheng, Shuli Liang, Shuangyan Han and Ying Lin

Table S1. Concentration of individual contaminant used in single toxicity test.

Contaminants	Concentration (mg/l)	
	Acute test	Chronic test
BPA	0.047–9.375	0.45–45
Cr	0.188–46.875	0.90–45
Cd	0.938–56.250	0.90–45
Pb	0.938–46.875	\
As	0.188–46.875	0.9–45
Hg	0.023–2.813	0.45–45
Ni	0.375–14.063	\

Table S2. Concentrations of individual contaminants in stocked mixtures.

Contaminants	Ratio	Concentration	
		BPA	Heavy metals
BPA+Cr	1:1	$2.5EC_{50}$	$2.5EC_{50}$
BPA+Pb	1:1	$2.5EC_{50}$	$2.5EC_{50}$
BPA+Hg	1:1	$2.5EC_{50}$	$2.5EC_{50}$
BPA+Ni	1:1	$2.5EC_{50}$	$2.5EC_{50}$
BPA+Cd	$10^{-1.5}$:1	$0.152EC_{50}$	$4.848EC_{50}$
BPA+Cd	10^{-1}:1	$0.455EC_{50}$	$4.545EC_{50}$
BPA+Cd	$10^{-0.5}$:1	$1.19EC_{50}$	$3.81EC_{50}$
BPA+Cd	1:1	$2.5EC_{50}$	$2.5EC_{50}$
BPA+Cd	$10^{0.5}$:1	$3.81EC_{50}$	$1.19EC_{50}$
BPA+Cd	10^{1}:1	$4.545EC_{50}$	$0.455EC_{50}$
BPA+Cd	$10^{1.5}$:1	$4.848EC_{50}$	$0.152EC_{50}$
BPA+As	$10^{-1.5}$:1	$0.152EC_{50}$	$4.848EC_{50}$
BPA+As	10^{-1}:1	$0.455EC_{50}$	$4.545EC_{50}$
BPA+As	$10^{-0.5}$:1	$1.19EC_{50}$	$3.81EC_{50}$
BPA+As	1:1	$2.5EC_{50}$	$2.5EC_{50}$
BPA+As	$10^{0.5}$:1	$3.81EC_{50}$	$1.19EC_{50}$
BPA+As	10^{1}:1	$4.545EC_{50}$	$0.455EC_{50}$
BPA+As	$10^{1.5}$:1	$4.848EC_{50}$	$0.152EC_{50}$

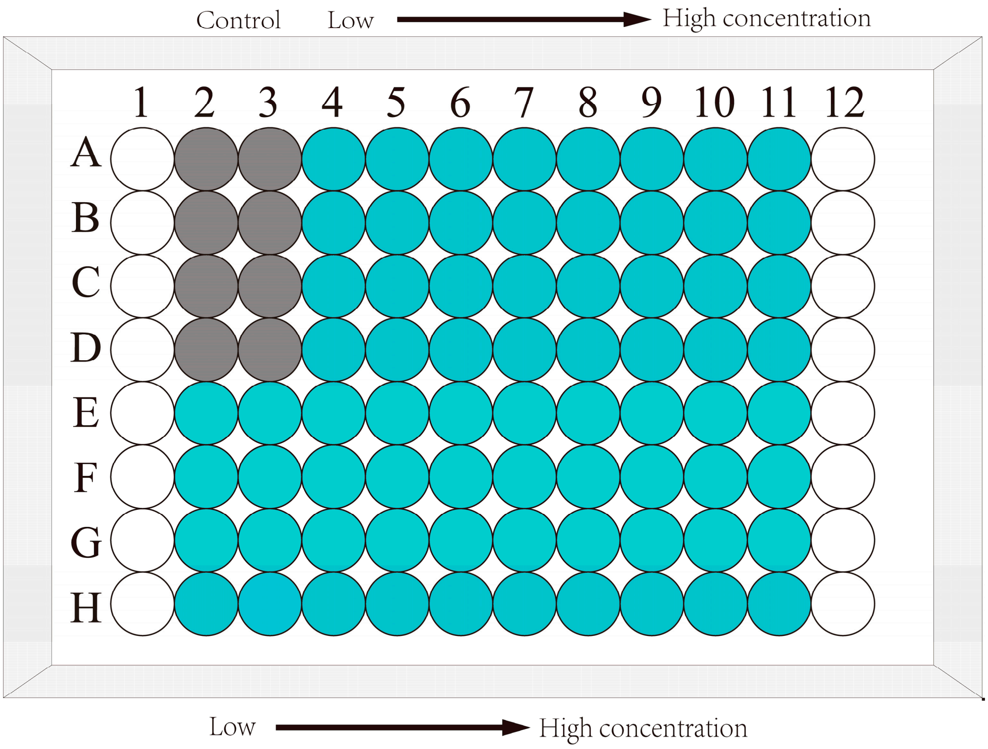

Figure S1. Arrangement of control and test samples in 96-well microplate. Gray solid circles are the control, and the blue solid circles are test samples. Each concentration of sample locates in four wells in same column.

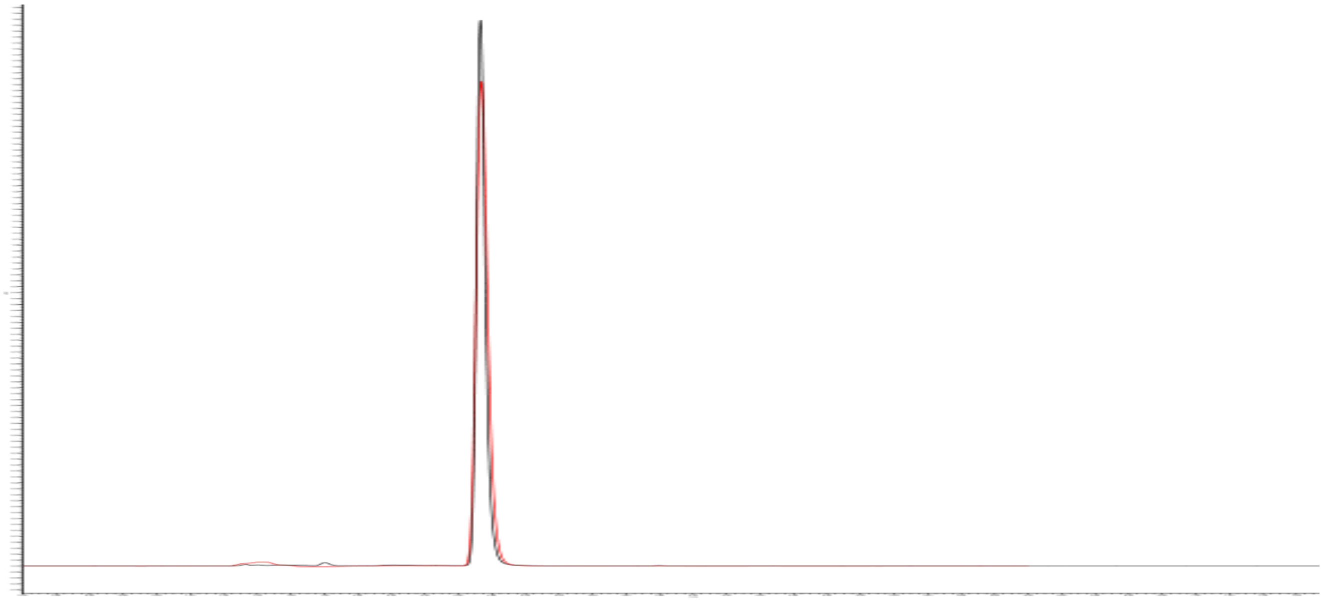

Figure S2. Comparison of aqueous BPA solution and methanol dissolved BPA using HPLC. Black line, methanol dissolved BPA solution. Red line, aqueous BPA solution.

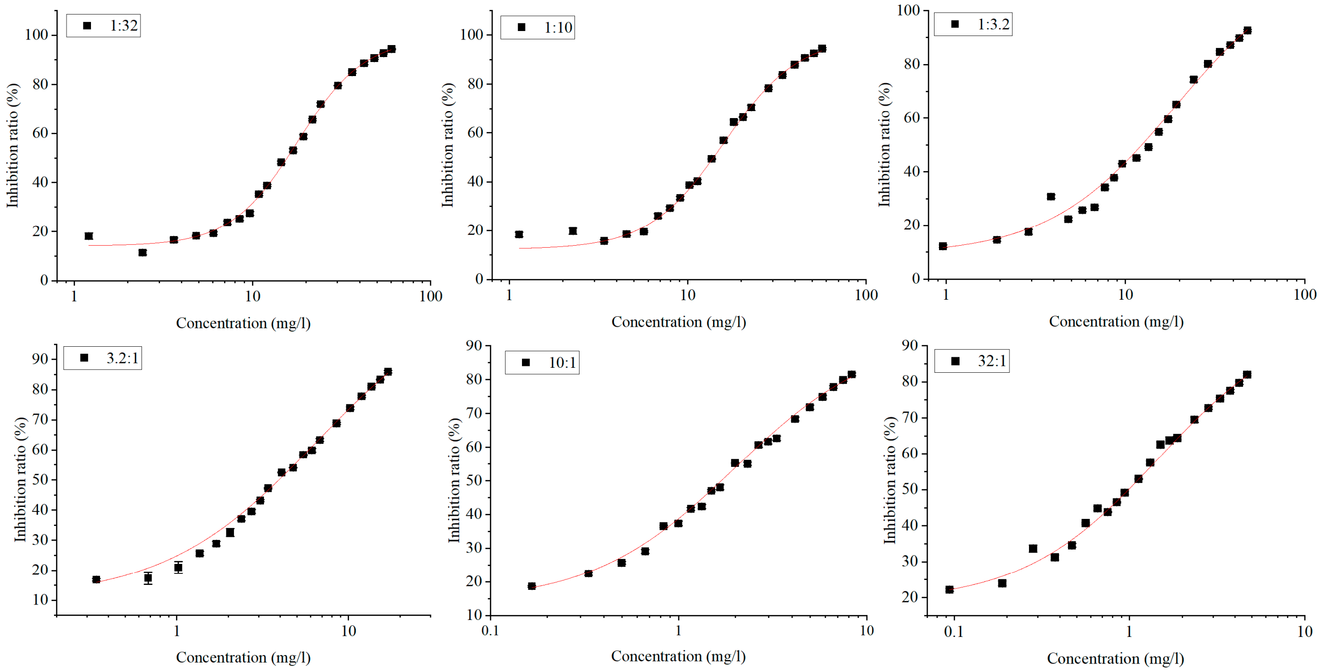

Figure S3. Observed acute mixture toxicity of BPA and Cd at non−equitoxic ratio.

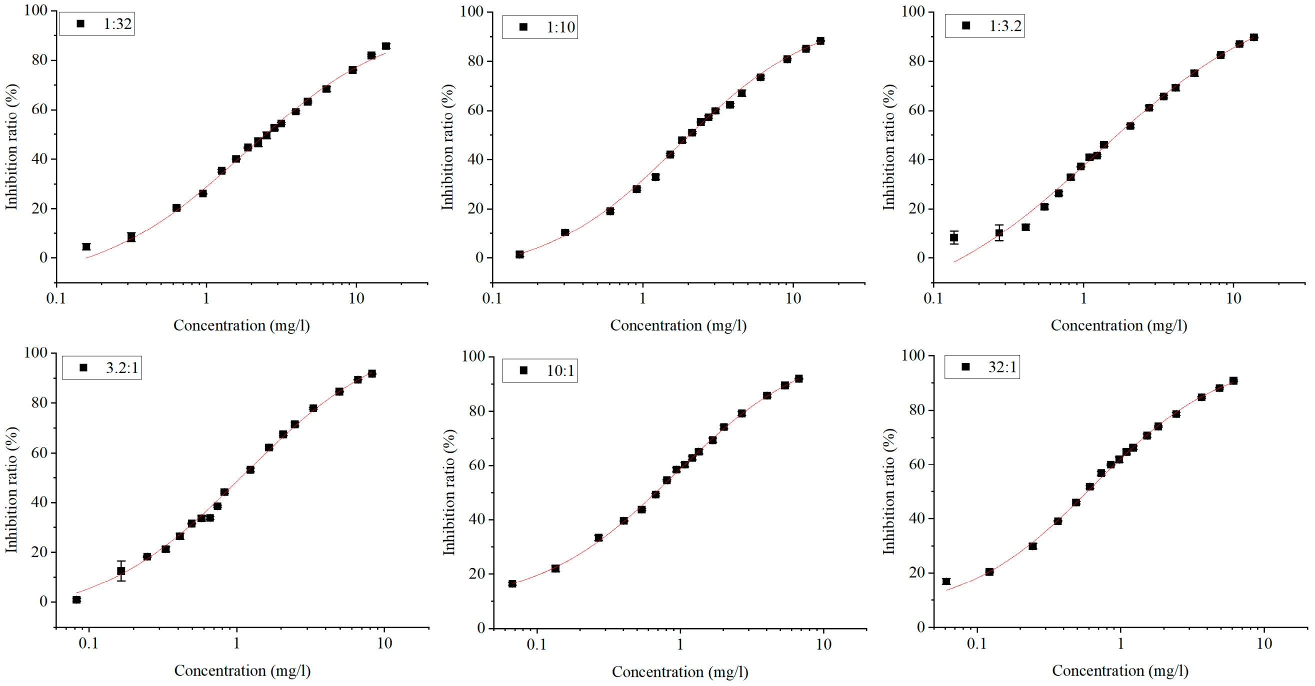

Figure S4. Observed acute mixture toxicity of BPA and As at non−equitoxic ratio.

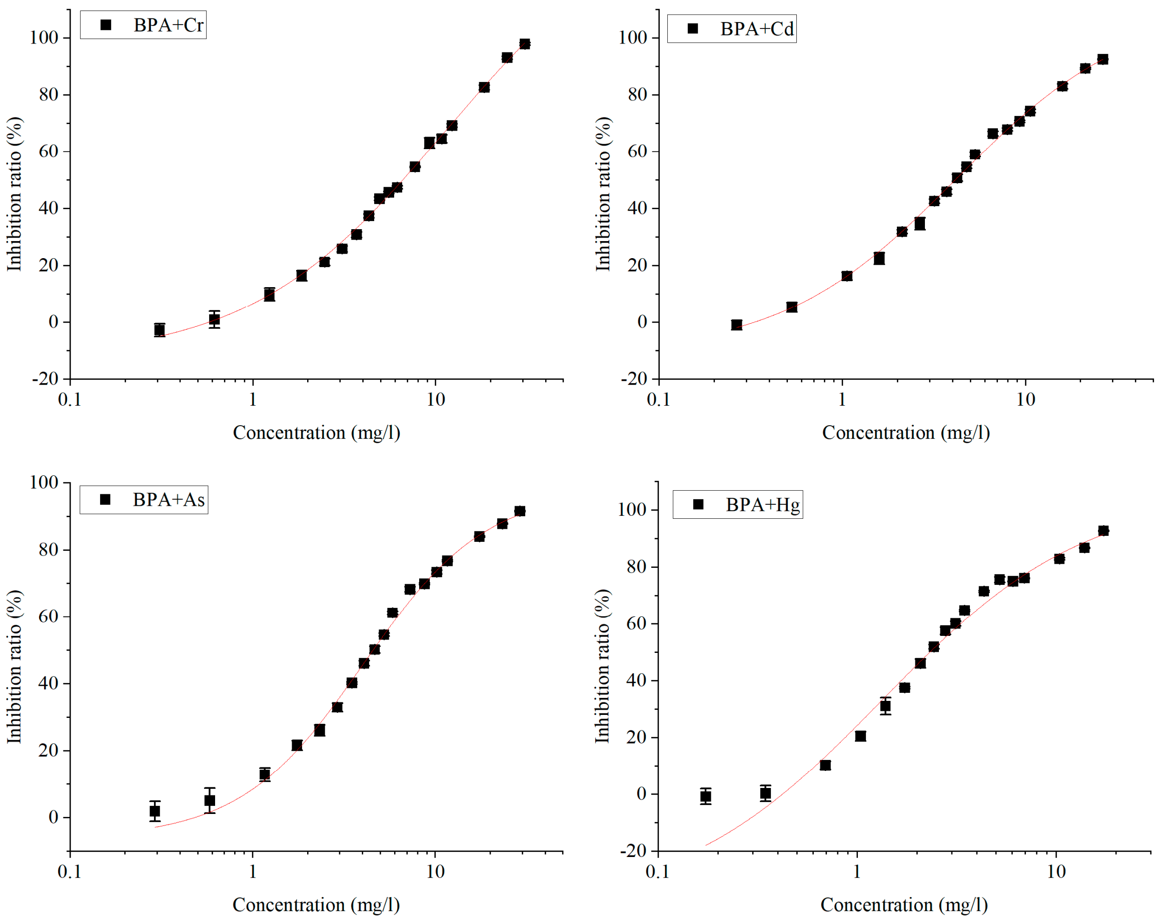

Figure S5. Observed chronic mixture toxicity of BPA and heavy metals at equitoxic ratio.

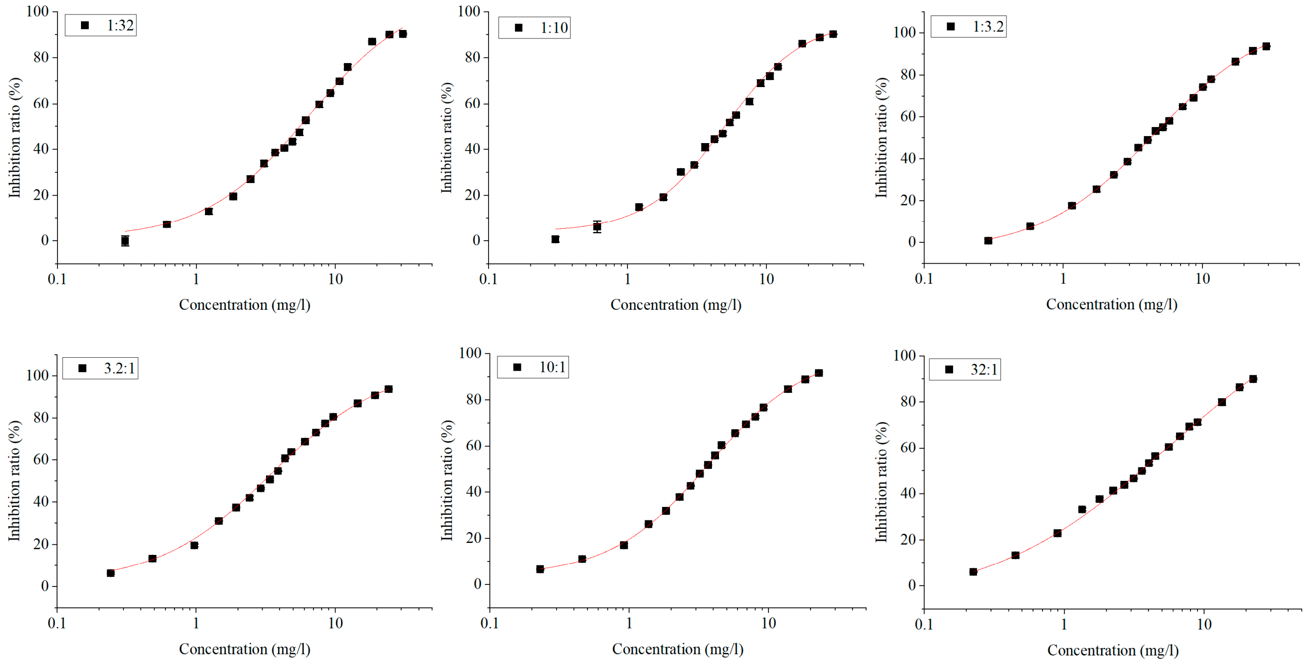

Figure S6. Observed chronic mixture toxicity of BPA and Cd at non−equitoxic ratio.

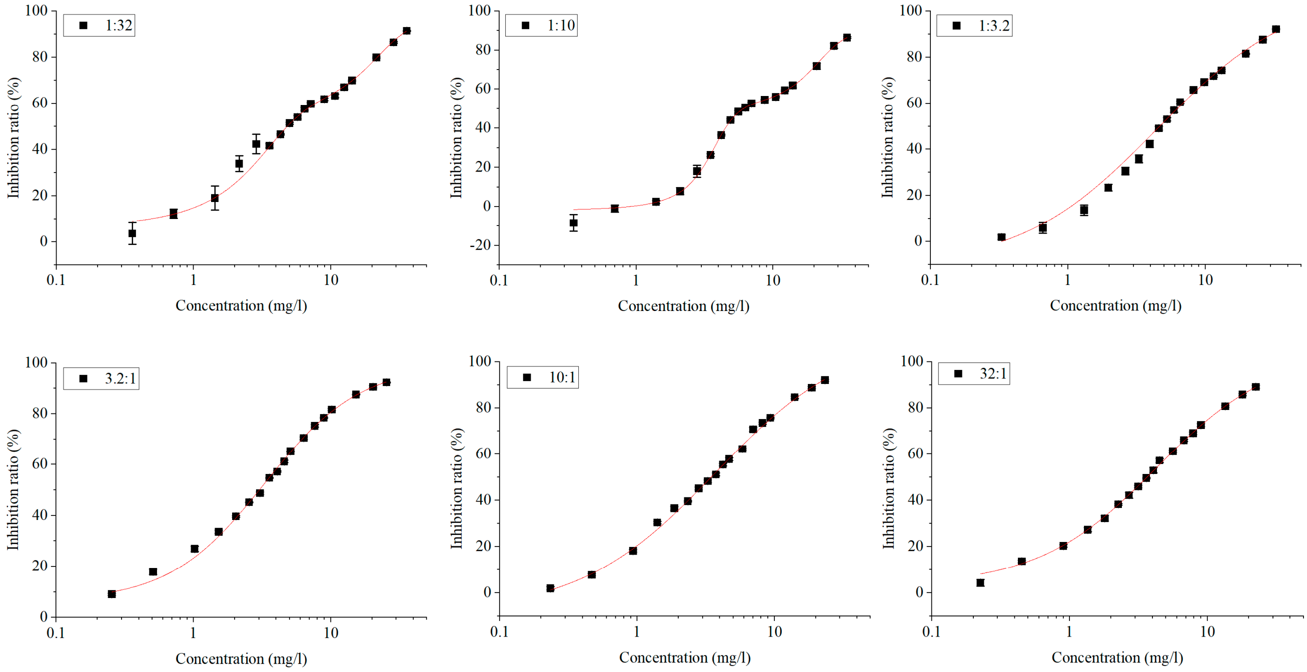

Figure S7. Observed chronic mixture toxicity of BPA and As at non−equitoxic ratio.
